# Genomic insight into *Campylobacter jejuni* isolated from commercial turkey flocks in Germany using whole-genome sequencing analysis

**DOI:** 10.3389/fvets.2023.1092179

**Published:** 2023-02-16

**Authors:** Hosny El-Adawy, Helmut Hotzel, Silvia García-Soto, Herbert Tomaso, Hafez M. Hafez, Stefan Schwarz, Heinrich Neubauer, Jörg Linde

**Affiliations:** ^1^Institute of Bacterial Infections and Zoonoses, Friedrich-Loeffler-Institut, Jena, Germany; ^2^Faculty of Veterinary Medicine, Kafrelsheikh University, Kafr El-Sheikh, Egypt; ^3^Institute of Poultry Diseases, Free University Berlin, Berlin, Germany; ^4^Institute of Microbiology and Epizootics, Centre for Infection Medicine, Department of Veterinary Medicine, Freie Universität Berlin, Berlin, Germany; ^5^Veterinary Centre of Resistance Research (TZR), Freie Universität Berlin, Berlin, Germany

**Keywords:** *Campylobacter jejuni*, turkeys, genetic diversity, WGS, MLST, resistome, virulome

## Abstract

*Campylobacter* (*C*.) *jejuni* is a zoonotic bacterium of public health significance. The present investigation was designed to assess the epidemiology and genetic heterogeneity of *C. jejuni* recovered from commercial turkey farms in Germany using whole-genome sequencing. The Illumina MiSeq^®^ technology was used to sequence 66 *C. jejuni* isolates obtained between 2010 and 2011 from commercial meat turkey flocks located in ten German federal states. Phenotypic antimicrobial resistance was determined. Phylogeny, resistome, plasmidome and virulome profiles were analyzed using whole-genome sequencing data. Genetic resistance markers were identified with bioinformatics tools (AMRFinder, ResFinder, NCBI and ABRicate) and compared with the phenotypic antimicrobial resistance. The isolates were assigned to 28 different sequence types and 11 clonal complexes. The average pairwise single nucleotide-polymorphisms distance of 14,585 SNPs (range: 0–26,540 SNPs) revealed a high genetic distinction between the isolates. Thirteen virulence-associated genes were identified in *C. jejuni* isolates. Most of the isolates harbored the genes *fla*A (83.3%) and *fla*B (78.8%). The *wla*N gene associated with the Guillain–Barré syndrome was detected in nine (13.6%) isolates. The genes for resistance to ampicillin (*bla*_OXA_), tetracycline [*tet*(O)], neomycin [*aph*(3')-IIIa], streptomycin (*aadE*) and streptothricin (*sat4*) were detected in isolated *C. jejuni* using WGS. A gene cluster comprising the genes *sat4, aph*(3′)-IIIa and *aadE* was present in six isolates. The single point mutation T86I in the housekeeping gene *gyrA* conferring resistance to quinolones was retrieved in 93.6% of phenotypically fluoroquinolone-resistant isolates. Five phenotypically erythromycin-susceptible isolates carried the mutation A103V in the gene for the ribosomal protein L22 inferring macrolide resistance. An assortment of 13 β-lactam resistance genes (*bla*_OXA_ variants) was detected in 58 *C. jejuni* isolates. Out of 66 sequenced isolates, 28 (42.4%) carried plasmid-borne contigs. Six isolates harbored a pTet-like plasmid-borne contig which carries the *tet*(O) gene. This study emphasized the potential of whole-genome sequencing to ameliorate the routine surveillance of *C. jejuni*. Whole-genome sequencing can predict antimicrobial resistance with a high degree of accuracy. However, resistance gene databases need curation and updates to revoke inaccuracy when using WGS-based analysis pipelines for AMR detection.

## 1. Introduction

*Campylobacter* (*C*.) is recognized as the leading cause of bacterial gastroenteritis in humans and several animal species (poultry, cattle, pigs, sheep and goats) worldwide. *C. jejuni* is a commensal bacterium of animal species and, therefore, exposed to antimicrobial agents that are administered to animals for various reasons. Moreover, the environment can be contaminated with *Campylobacter* by litter and/or soil at farm premises (1). Poultry and their products are considered the most significant source of human campylobacteriosis (2, 3). As a consequence, antimicrobial resistance in *C. jejuni* is a growing problem (4, 5). Some clones of *C. jejuni* endure genetically stable over long periods of time, but *C. jejuni* can adapt to different environmental conditions by means of variation in the isolate's virulence (6).

In Germany, the prevalence of *Campylobacter* in poultry meat and chickens from 2001 through 2010 ranged from 14 to 34% and 6 to 64% per year, respectively (7). A voluntary monitoring program was conducted between 2004 and 2007 in broiler farms and the reported human incidence in Germany in order to identify the prevalence patterns of thermotolerant *Campylobacter* spp. showed that the peak in human campylobacteriosis preceded the peak in broiler prevalence in Lower Saxony (8). *C. jejuni* was isolated from different poultry species in Germany and showed a higher prevalence than *C. coli* (9).

It is necessary to understand virulence factors and molecular mechanisms contributing to pathogenesis of *Campylobacter*. Whole-genome sequences can be used for high-resolution genotyping and automatized detection of genetic markers for virulence, antimicrobial resistance and mobile genetic elements (10–12). Since the costs for WGS are decreasing, it has replaced traditional typing methods, such as pulsed-field gel electrophoresis (PFGE), multi-locus sequence typing (MLST) and serotyping for surveillance of bacterial infectious diseases by public health authorities (11–19). Consequently, WGS was also demonstrated for the investigation of virulence, clonality and antimicrobial resistance in *Campylobacter* isolated from poultry farms (20–23).

The WGS led to the creation of the core genome multi-locus sequence typing (cgMLST), a typing method encompassing hundreds of loci from the traditional seven loci of MLST (24). Additionally, studies using single nucleotide polymorphism (SNP) allow the establishment of the best phylogenetic relationship among different pathogens (25). The WGS is used for various purposes including novel antibiotic and diagnostic test development, studying the emergence of antibiotic resistance, disease surveillance, and direct infection control measures in both clinical settings and communities (26). The next-generation sequencing (NGS) systems available include Illumina Genome Analyzer (HiSeq, MiSeq), Life Technologies Ion Torrent, and the PacBio RX system (27).

The used WGS data revealed a high genetic diversity amongst *C. jejuni* isolated from broilers and definite types and virulence genes are implicated with the development of more severe human illness (28).

In Europe, the antimicrobial resistance of *C. jejuni* isolated from chickens and turkeys had to be reported every 2 years based on European Union Commission Implementing Decision 2013/652/EU (29). In Germany, the antimicrobial resistance of *Campylobacter* spp. isolated from broilers and turkeys was highest to ciprofloxacin, nalidixic acid and tetracycline whereas *C. coli* were more often resistant than *C. jejuni* and resistance was observed more frequently in turkeys than in broilers (30). The emergence of a high antimicrobial resistance and multidrug resistance was identified in *C. jejuni* isolated from commercial turkey farms in Germany (31).

The objective of this study was to analyze *C. jejuni* isolated from turkey flocks using WGS for high-resolution genotyping and to investigate their complete genomic potential concerning resistance to antimicrobial agents, plasmids and virulence-associated factors.

## 2. Materials and methods

### 2.1. Bacterial isolates and growth conditions

Sixty-six *C. jejuni* were isolated from 66 turkey flocks reared in different turkey farms in ten federal states in Germany, namely Baden-Wuerttemberg, Bavaria, Brandenburg, Mecklenburg-Western Pomerania, Lower-Saxony, North Rhine-Westphalia, Rhineland-Palatinate, Saxony, Saxony-Anhalt and Thuringia. The samples were collected from apparently healthy turkey flocks aged between the 12th and the 18th weeks (Table S1). The isolation was carried out according to ISO 10272 (32). All isolates were identified using MALDI-TOF MS and multiplex PCR assay as described previously by El-Adawy et al. (31).

### 2.2. Antimicrobial susceptibility testing

The broth microdilution test was performed using commercially available microtitre plates TREK^®^ Sensititre NLDMV2 (Trek Diagnostic Systems, Ltd., East Grinstead, UK) for the determination of the antimicrobial susceptibility of the 66 *C. jejuni* isolates to gentamicin, chloramphenicol, streptomycin, erythromycin, neomycin, amoxicillin, tetracycline, nalidixic acid, ciprofloxacin and metronidazole. The susceptibility test was performed according to CLSI recommendations and the plates were incubated under microaerophilic condition (CampyGen™, Oxoid Deutschland GmbH, Schwerte, Germany) at 37°C for 48 h (33). The results were read either visually or photometrically (Tecan Deutschland GmbH, Crailsheim, Germany) using the computer program easyWIN fitting (version V6.1, 2000; Tecan Deutschland GmbH, Crailsheim, Germany). *C. jejuni* ATCC 33560 (American Type Culture Collection, LGC Standards GmbH, Wesel, Germany) was used as reference strain for quality control in each batch of the broth microdilution tests. The resistance breakpoints for gentamicin, chloramphenicol, erythromycin, amoxicillin, tetracycline, nalidixic acid and ciprofloxacin were those recommended by the Clinical and Laboratory Standards Institute (33, 34) and in previously published literature (31, 35). The resistance breakpoint used for streptomycin was ≥ 64 μg/ml, as described previously (35). Since there were no CLSI breakpoints for neomycin, we used a tentative breakpoint for *Escherichia coli* of 32 μg/mL (36, 37). *C. jejuni* isolates were tested for resistance to metronidazole and the breakpoint for resistance at 16 mg/ml (38) was used.

### 2.3. DNA extraction for WGS analysis

Genomic DNA was extracted and purified from a 48 h bacterial culture on Mueller-Hinton blood agar plates (Oxoid Deutschland GmbH, Wesel, Germany) using QIAGEN^®^ Genomic-tip 20/G Kit (QIAGEN^®^, Hilden, Germany) according to the manufacturer's instructions. The DNA was eluted in 200 μl elution buffer. DNA was quantified spectrophotometrically using a Nanodrop^®^ ND-1000 (Fisher Scientific GmbH, Schwerte, Germany). The quality of the DNA was determined using the Qubit dsDNA BR Assay Kit (Invitrogen, Carlsbad CA, USA).

### 2.4. Whole-genome sequencing

Sequencing libraries were created using the Nextera XT DNA Library Preparation Kit (Illumina Inc., San Diego, CA). Paired-end sequencing was performed on an Illumina MiSeq instrument according to the manufacturer's instructions (Illumina Inc., San Diego, CA) producing 300 bp long reads. Raw sequencing data were deposited by the European Nucleotide Archive (ENA) under the BioProject PRJEB55640. The bioinformatic analysis started with quality control of the raw paired-end reads from Illumina. The Linux-based bioinformatics pipeline WGSBAC v. 2.0.0 (https://gitlab.com/FLI_Bioinfo/WGSBAC) uses FastQC v. 0.11.7 (39) and calculates coverage of raw reads. Second, WGSBAC performs assembly using Shovill v. 1.0.4 (https://github.com/tseemann/shovill). To check the quality of the assembled genomes, WGSBAC uses QUAST v. 5.0.2 (40) and to identify potential contamination on both reads and assemblies, the pipeline uses Kraken 2 v. 1.1 (41) and the database Kraken2DB. For the investigation of antimicrobial resistance genes and virulence determinants, WGSBAC uses the software ABRicate (v. 0.8.10) (https://github.com/tseemann/abricate) and the databases: ResFinder (42), NCBI (43) and Virulence Factor Database (VFDB) (44). In addition, WGSBAC uses AMRFinderPlus (v. 3.6.10) (45) for the detection of chromosomal point mutations leading to AMR and organism-specific acquired resistance genes. For plasmid detection, Platon was used (46). BLAST search was performed with plasmid-borne contigs against NCBI's nucleotide database and hits were compared to recently published pTet-like plasmids (47, 48).

Annotation of the assembled genomes was performed by the software Bakta v. 1.6.1 (https://github.com/oschwengers/bakta) (49). Annotated features of interest were visualized using the software Geneious Prime v. 2021.0.1 (https://www.geneious.com).

For genotyping, WGSBAC uses classical multilocus sequence typing (MLST) on assembled genomes using the software mlst v. 2.16.1 that incorporates the PubMLST database for the seven gene *C. jejuni*/*coli* MLST scheme (https://pubmlst.org/organisms/campylobacter-jejunicoli). Core genome multilocus sequence typing was performed using the external software Ridom Seqsphere+ v. 5.1.0 with default settings and the specific core genome scheme (cgMLST v2). In addition, WGSBAC performs mapping-based genotyping using core-genome single nucleotide polymorphisms (cgSNPs) identified by Snippy v. 4.3.6 (https://github.com/tseemann/snippy) with standard settings. As reference the genome of *C. jejuni* NCTC 11168 (accession NC_002163.1) was used. For phylogenetic tree construction based on cgSNP analysis, WGSBAC uses the SNPs alignment matrix generated by Snippy and reconstructs the tree using RAxML (Randomized Axelerated Maximum Likelihood) v. 8 (50). The tree was rooted to the reference genome and visualized using the interactive Tree of Life (iTOL) v. 4 web tool (https://itol.embl.de/login.cgi).

## 3. Results

### 3.1. Phylogenetic analysis and MLST/cgMLST analysis

Sequencing of the 66 *C. jejuni* isolates, yielded an average of 807,061 total reads (range: 156,702–2,263,714) per sample, with an average read length of 219 bp (Table S1) leading to an average read-coverage of 104-fold (range: 37–139). The assembled genomes consisted on average of 29 contigs (range: 20–256). The GC content was 30.43% and the genome size of the isolates was 1,700,350 bp on average (range: 1,410,342–1,883,846 bp).

**Figure 1:**
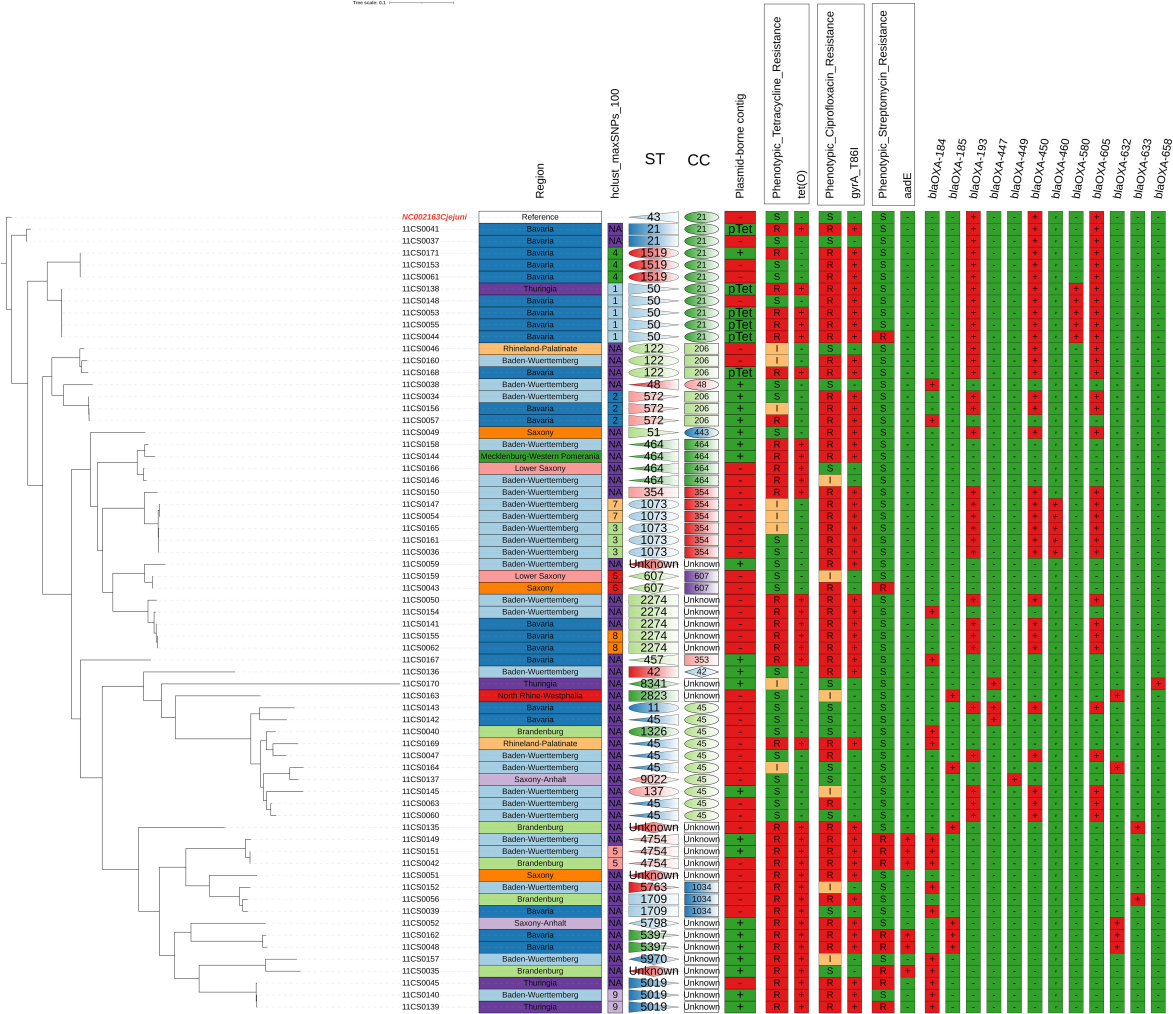
Phylogentic and genotyping analysis using cgMSLT (Phylogenetic trees based on cgSNP distances) rooted by the most distant isolate. Comparison between phenotypic and genotypic antimicrobial resistance of the tested 66 *C. jejuni* isolates with regard to the place of isolation and sequence types: correlation of susceptibility phenotypes and genotypes.

The phylogentic and genotyping analysis displaying relatedness between the 66 *C. jejuni* isolates using cgMSLT based on cgSNP distances rooted to the reference genome revealed nine different groups according to how closely related the isolates were (Figure 1).

**Figure 2:**
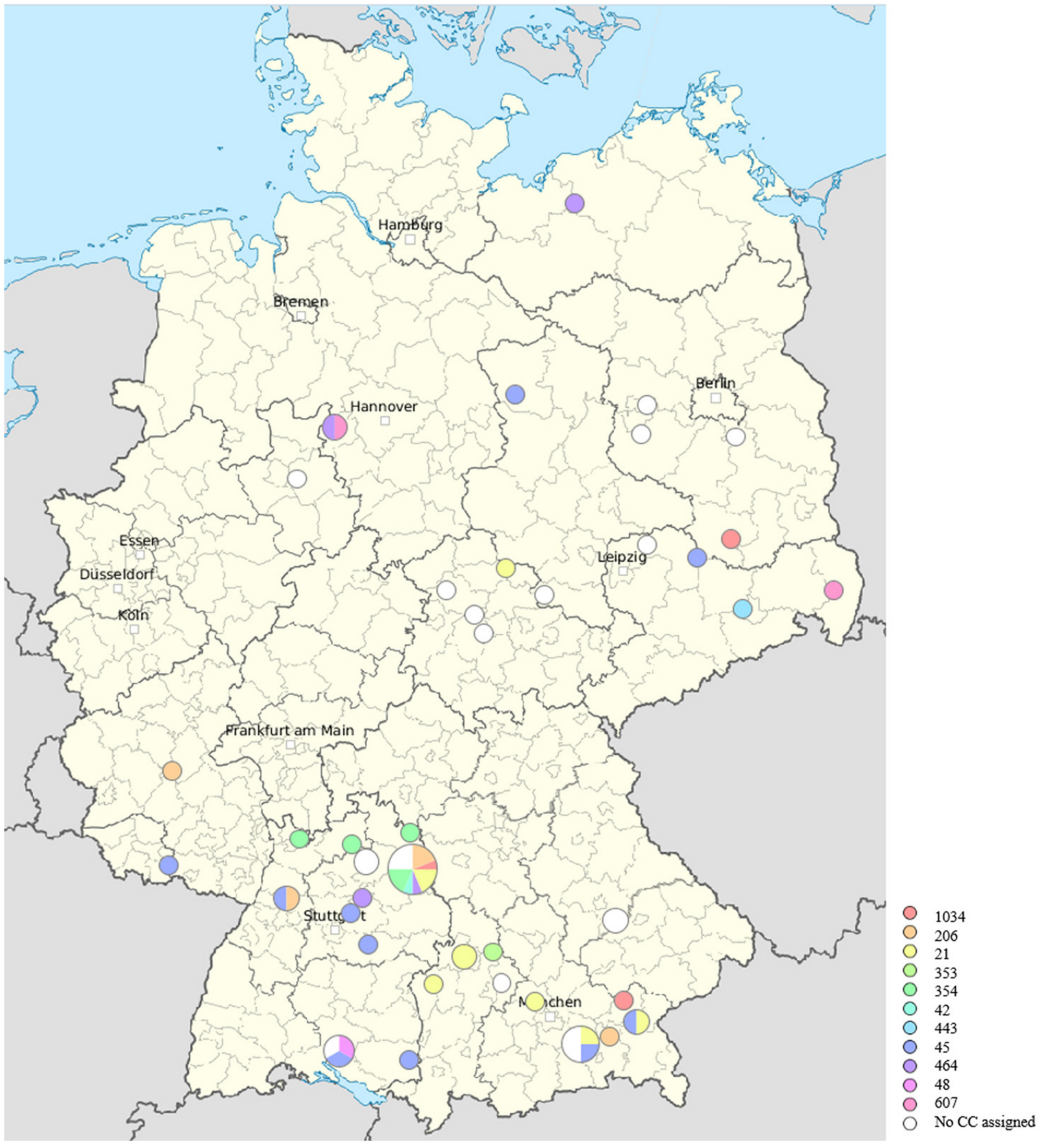
Origin of the 66 *C. jejuni* and their clonal complexes in 11 federal states in Germany.

The origin and distribution of the 66 *C. jejuni* and their colonal complexes in 11 federal states in Germany were shown in Figure 2.

The MLST analysis based on WGS revealed a high genetic diversity with 28 different sequence types (STs). For four isolates, a so far unknown MLST type was detected. The most prevalent STs found were ST 45 (9%), ST 50 (8%), ST 1073 (8%), ST 2274 (8%), and ST 464 (6%) (Table S1, Figure 1). The high genetic diversity was further indicated by an average core-gene Single Nucleotide-Polymorphisms distance of 14,585 cgSNPs (range 0–26,540 cgSNPs). Hierarchical clustering with a cut-off of 100 cgSNPs grouped 29 isolates into nine clusters, while the majority of isolates did not cluster. Finally, cgMLST analysis supported the finding of high genetic diversity, as 11 different clonal complexes (CCs) were detected among the 66 isolates (Figure 1, Table S1). For four isolates, an unknown clonal complex was identified. Both the SNP-based phylogeny and the cgMLST analysis indicated the genetic relatedness of isolates partly following the geographical origins from where they were isolated (Figures 1, 2). While the genetic diversity was generally high, some isolates were closely related (Figure 1, Table S1). For example, isolates 11CS0055 and 11CS0044 from Bavaria were indistinguishable (0 cgSNPs).

The two isolates 11CS0036 and 11CS0161 from samples obtained in Baden-Wuerttemberg were closely related and revealed only one cgSNP (Table S1), while other isolates (11CS0043 and 11CS0159) collected from two different federal states (Saxony and Lower Saxony) had two SNPs difference. Three SNPs difference was found in two isolates (11CS0042 and 11CS0151) from Baden-Wuerttemberg. Four isolates from Bavaria (11CS0061, 11CS0171, 11CS0062, and 11CS0155) seemed to be closely related with six SNPs difference (Table S1).

### 3.2. Determination of virulence-associated genes

The genomic analysis of 66 *C. jejuni* isolates revealed in total 30 virulence-associated genes related to motility, chemotaxis, adhesion and invasion (Table 1, Table S1). Most of the isolates harbored the genes *fla*A (83.3%) and *fla*B (78.8%) coding for flagellin protein A and B, respectively. Among other virulence determinants, the gene *cj1135* coding for the putative two-domain glucosyltransferase was present in 56% of the isolates. The gene *rfbC* coding for dTDP-4-dehydrorhamnose 35-epimerase was detected in 42.4% and the motility accessory factor PseD/Maf2 in 28.8% of the *C. jejuni* isolates. Genes for sialic acid synthase (*neuB1*) and UDP-N-acetylglucosamine 2-epimerase (*neuC1*) were detected in 16.6% of the isolates. In minor proportion, the combination of *cj1136*-*138, neuA* and *cstIII* genes was found in 15.2% of the *C. jejuni* isolates. Out of the 66 *C. jejuni* isolates, eight (12.1%) isolates harbored type IV secretion system genes (*virB10, virB11, virB4, virB8, virB9, virD4* and *cjp54*). Seven isolates (10.6%) harbored *cj*421c, *cj*426c, *cj1432c, cj1435c, cj1436c, cj1437c, cj1440c, virB10, virB11, virB4, virB8, virB9, virD4*, and *cjp54* genes (Table 1). The *wlaN* gene associated with the Guillain–Barré syndrome was identified in nine (13.6%) isolates.

**Table 1:** Frequency of most significant virulence-associated genes found in in 66 *Campylobacter jejuni* isolates.

**Virulence gene**	**Name of protein**	**No. isolates positive (%)**
*fla*A	Flagellin	55 (83.3)
*fla*B	Flagellin	52 (78.8)
*cj*1135	Putative two-domain glucosyltransferase	37 (56.0)
*rfb*C	dTDP-4-dehydrorhamnose 35-epimerase	28 (42.4)
*maf*4	Motility accessory factor	19 (28.8)
*pse*D*-maf*2	Motility accessory factor PseD	19 (28.8)
*fcl*	GDP-L-fucose synthetase	12 (18.2)
*neu*B1	Sialic acid synthase	11 (16.7)
*neu*C1	UDP-N-acetylglucosamine 2-epimerase	11 (16.7)
*cj*1136	Putative glucosyltransferase	10 (15.2)
*cj*1137c	Putative glucosyltransferase	10 (15.2)
*cj*1138	Putative glucosyltransferase	10 (15.2)
*neu*A1	Bifunctional beta-14-N-acetylgalactosaminyltransferase/CMP-Neu5Ac synthase	10 (15.2)
*cst*III	Alpha-23 sialyltransferas	10 (15.2)
*wla*N	Beta-13 galactosyltransferase	9 (13.6)
*vir*B10	Type IV secretion system protein VirB10	8 (12.1)
*vir*B11	Type IV secretion system protein VirB11	8 (12.1)
*vir*B4	Type IV secretion system protein VirB4	8 (12.1)
*vir*B8	Type IV secretion system protein VirB8	8 (12.1)
*vir*B9	Type IV secretion system protein VirB9	8 (12.1)
*vir*D4	Type IV secretion system protein VirD4	8 (12.1)
*cjp*54	Type IV secretion system protein VirB7	8 (12.1)
*kfi*D	UDP-glucose 6-dehydrogenase	7 (10.6)
*cj*421c	Sugar transferase	7 (10.6)
*cj*426c	Methyltransferase family protein	7 (10.6)
*cj*1432c	Sugar transferase	7 (10.6)
*cj*1435c	Phosphatase	7 (10.6)
*cj*1436c	Aminotransferase	7 (10.6)
*cj*1437c	Aminotransferase	7 (10.6)

### 3.3. Phenotypic antimicrobial resistance

The results of antimicrobial susceptibility testing showed that all isolates were susceptible to gentamicin, erythromycin and chloramphenicol. Resistance to streptomycin, neomycin, tetracycline, nalidixic acid, ciprofloxacin and metronidazole was detected in 10 (15.2%), 18 (27.3%), 36 (54.5%), 44 (66.7%), 47 (71.2%), and 49 (74.2%) isolates, respectively (Table S1).

### 3.4. Genotypic antimicrobial resistance

**Figure 3:**
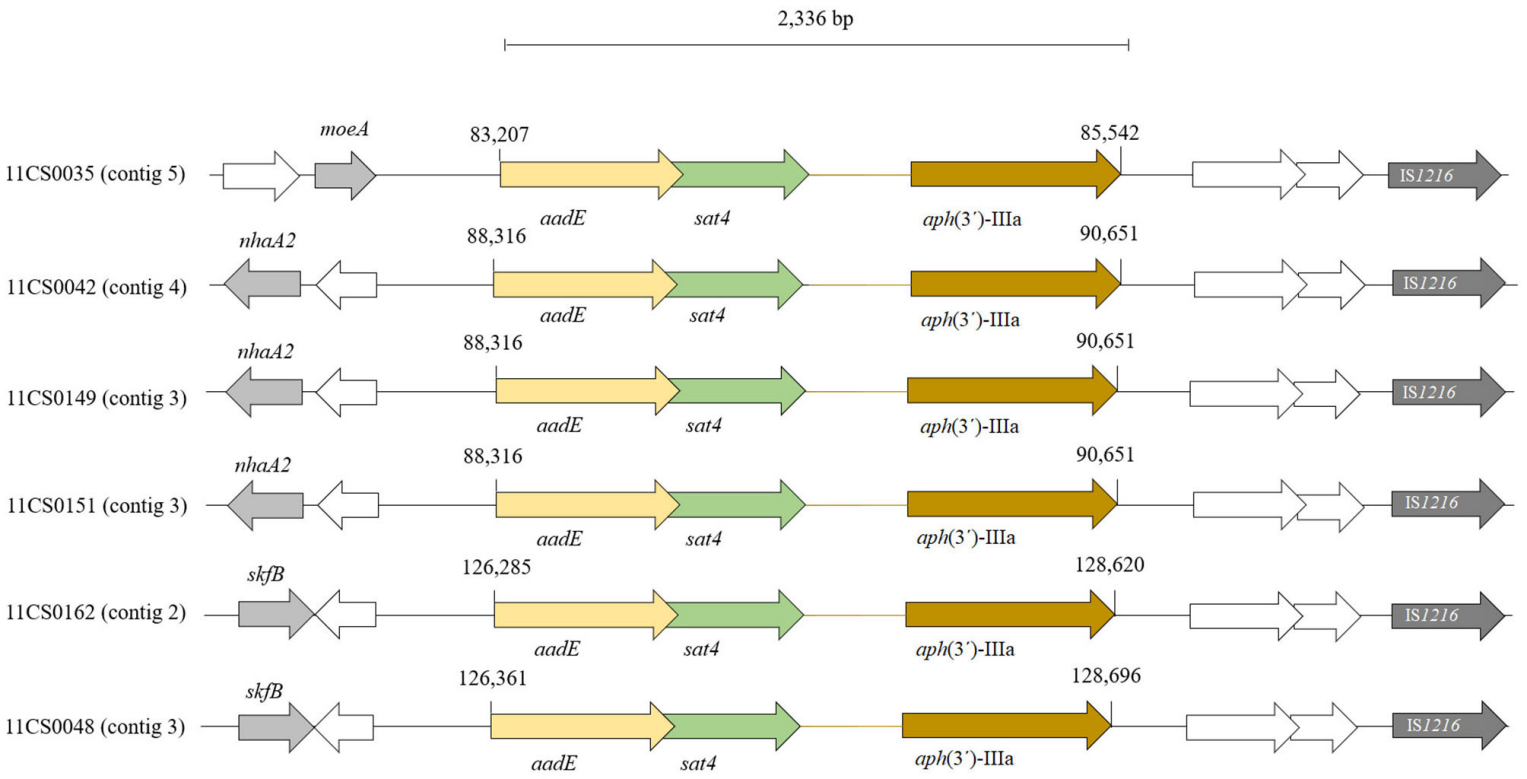
Distribution of *aadE, sat4* and *aph*(3')-IIIa gene cluster found in six *Campylobacter jejuni* isolates. ORFs in white indicate unnamed genes.

WGS analyses identified 18 acquired AMR genes that code for resistance to antimicrobials representing three different classes (tetracyclines, aminoglycosides and β-lactams) and point mutations in the *gyrA* gene coding for resistance to (fluoro) quinolones. The mutation associated with macrolide resistance was located in the gene for the ribosomal protein L22 (A103V) (Table 2, Table S1). The antimicrobial resistance gene associated with tetracycline resistance *tet*(O) was identified in 34 of the 66 *C. jejuni* isolates (51.5%) (Table 2, Table S1). Genes coding for aminoglycoside-modifying enzymes of two distinct families, aminoglycoside phosphotransferases (APHs) and aminoglycoside nucleotidyl transferases (ANTs) were detected. streptomycin resistance is encoded by *aadE* gene which was found in six out of ten (60.0%) phenotypically streptomycin-resistant *C. jejuni*. Out of 18 *C. jejuni* isolates resistant to neomycin, the *aph*(3′)-IIIa gene was detected in six (33.3%) isolates (Table 2). The *sat4* gene encoding streptothricin resistance was detected in six (9.1%) *C. jejuni* isolated in this study. A gene cluster comprising the genes *sat4, aph*(3′)-IIIa and *aadE* was present in six isolates (Figure 3). Upstream the gene cluster, all the isolates have two ORFs, one codes for the HTH domain-containing protein and the other for a hypothetical protein. They are followed by the IS*6*-like element IS*1216* family transposase. Downstream, variations within the isolates were detected: isolate 11CS0035 has the gene *moeA1* that codifies for the product molybdopterin molybdenumtransferase MoeA and an unnamed gene that codes for the AraC family transcriptional regulator. Downstream of the gene cluster, in isolates 11CS0042, 11CS0149 and 11CS0151, an unnamed gene was found that codes for the putative motility protein and the gene *nhaA2* that codes for the Na^+^/H^+^ antiporter NhaA. Isolates 11CS0162 and 11CS0048 have downstream an unnamed gene that codes for an ABC transporter substrate-binding protein and the gene *skfB* for the radical SAM protein.

**Table 2:** Distribution of antimicrobial resistance genes/mutations in the tested 66 *Campylobacter jejuni* isolated using different tools of analysis and compatibility with phenotypic resistance.

**Antimicrobial class**	**Antimicrobial agent**	**Phenotypic AMR**	**AMR gene or mutation**	**No. of positive isolates by WGS** ^*^			
				**AMR-Finder**	**ResFinder**	**NCBI**	**CARD**
Tetracyclines	Tetracycline	36	*tet*(O)	34	28	34	34
Fluoroquinolones	Ciprofloxacin	47	*gyr*A T86I	44	–	–	–
ß-Lactams			*bla*_OXA−184_	14	16	–	–
			*bla*_OXA−185_		6	6	6
			*bla*_OXA−193_	21	31		
			*bla*_OXA−447_	2	3	3	3
			*bla*_OXA−449_	1	1	1	1
			*bla*_OXA−450_				31
			*bla*_OXA−460_	5	–	–	–
			*bla*_OXA−465_	1	2	1	2
			*bla*_OXA−580_	5	–	–	–
			*bla*_OXA−605_		–	31	–
			*bla*_OXA−632_	3	–	5	–
			*bla*_OXA−633_		–	2	–
			*bla*_OXA−658_	1	–	-	–
Streptothricins	Streptothricin	–	*sat4*	6	–	6	6
Aminoglycosides	Streptomycin	10	*aadE*	–	6	–	6
	Neomycin	18	*aph*(3′)-IIIa	6	6	6	6

* The green highlighted data showed the agreement between different tools.

Twelve known variants of *bla*_OXA_ genes involved in β-lactam resistance were detected in this study using the results of different bioinformatic tools (AMRFinder, ResFinder, NCBI and ABRicate) (Table 2). Fifty-eight isolates harbored *bla*_OXA_ genes (52 resistant, five susceptible and one intermediate against ampicillin) (Table S2). Out of 61 phenotypically ampicillin-resistant *C. jejuni* isolates, 57 (93.4%) carried at least one gene coding for β-lactamases of the OXA-like family.

The distribution of detected 13 *bla*_OXA_ genes in 66 *C. jejuni* isolates is shown in Table S2 and Figure 1. In brief, *bla*_OXA−184_, *bla*_OXA−185_, *bla*_OXA−193_, *bla*_OXA−447_, *bla*_OXA−449_, *bla*_OXA−450_, *bla*_OXA−460_, *bla*_OXA−465_, *bla*_OXA−580_, *bla*_OXA−605_, *bla*_OXA−632_, *bla*_OXA−633_ and *bla*_OXA−658_-like genes were detected in 16 (24.2%), 6 (9.1%), 31 (47.0%), 3 (4.5%), 1 (1.5%), 31 (47.0%), 5 (7.6%), 2 (3.0%), 5 (7.6%), 31 (47.0%), 5 (7.6%), 2 (3.0%), 2 (3.0%), and 1 (1.5%) of 66 *C. jejuni* isolates, respectively (Table S2, Figure 1). Nineteen isolates harbored one gene coding for β-lactams of the OXA-like family (16 *bla*_OXA−184_, 1 *bla*_OXA−465_, 1 *bla*_OXA−447_ and 1 *bla*_OXA−449_. Twenty isolates carried three *bla*_OXA_ genes together (*bla*_OXA−193_, *bla*_OXA−450_ and *bla*_OXA−605_) while seven and 11 isolates harbored two and four *bla*_OXA_ genes, respectively (Table S2).

The accordance between phenotypic and genotypic antimicrobial resistance is shown in Table 2, Figure 1, and Table S1. Thirty-four (94.4%) out of 36 phenotypically tetracycline-resistant isolates harbored the *tet*(O) gene which confers tetracycline resistance (Table 2). Forty-seven (71.2%) *C. jejuni* isolates were phenotypically resistant to ciprofloxacin, 44 (93.6%) of them contained a chromosomal single point mutation in the *gyrA* gene which resulted in the amino acid substitutions C257T or 786I. All isolates carrying this mutation were resistant to (fluoro) quinolones (ciprofloxacin and nalidixic acid). Six isolates that were phenotypically intermediate to ciprofloxacin did not have any point mutation in the *gyrA* gene (Table 2). The *cme*ABCR multidrug efflux complex was present in all isolates.

Twenty-eight out of the 66 (42.4%) *C. jejuni* isolates contained at least one contig classified as plasmid-borne (Table S1). While the majority of those isolates (*n* = 18) contained one contig classified as plasmid-borne, isolates 11CS0052 and 11CS0059 contained five and four plasmid contigs, respectively, and another six isolates harbored two plasmids. The sizes of the plasmid-borne contigs detected by Platon ranged from 1,600 bp to 44,793 bp. Six isolates harbored a pTet-like plasmid-borne contig which carried the *tet*(O) gene and were phenotypically tetracycline-resistant. In the assembled genomes from six phenotypically tetracycline-resistant isolates, Platon identified a pTet-like plasmid-borne contig within a contig in which the *tet*(O) gene was also identified. BLAST search of the plasmid sequences in GenBank revealed that similar plasmids have been previously found in *Campylobacter* spp. Thirty-eight plasmid-borne contigs had a BLAST hit in *C. jejuni*, while for six, a hit in *C. coli* was found (Table S1).

The origin of the isolates, their phylogenetic relatedness, STs and association with antimicrobial resistance patterns are depicted in Figure 1. There is little correlation between genotypes and the numbers of AMR genes in the investigated isolates. Isolates assigned to STs 1073, 2274, 122, 5019, 4754, and 50 showed highly similar AMR gene profiles.

## 4. Discussion

Whole-genome sequencing is a promising tool in public health as it is able to identify sources and routes of infections, to investigate outbreaks with the highest resolution and thus to improve surveillance of *C. jejuni* (51). This investigation presents an application of WGS for assessing the epidemiology of *C. jejuni*, isolated from turkey farms in Germany.

In the current investigation, 30 virulence-associated genes were identified in *C. jejuni* isolates. Interestingly, nine (13.6%) of the isolates carried the *wlaN* gene implicated in the Guillain–Barré syndrome (GBS), a polyneuropathic disorder damaging the peripheral nervous system and causing muscle weakness (52). In north-eastern Spain *wlaN* was detected in two (16.6%) *C. jejuni* isolates from broilers (20). The gene *wlaN* was also detected in 10.7% of the tested *C. jejuni* isolated from slaughterhouses for broilers in Southern Brazil (53) and in 4.7% and 23.8% of isolates from broiler feces and poultry meat in Japan, respectively (54). Isolates harboring the *wlaN* gene may have a higher pathogenic potential and can induce autoimmune disease in their hosts (53). A previous study showed that the presence of the *wlaN* gene increased the capacity of cell invasion (*in vitro* and *in vivo*) (55).

This study revealed on the one hand a high genetic diversity of the analyzed 66 *C. jejuni* isolates indicated by 28 STs, 11 CCs and an average pairwise cgSNP distance of 14,585. On the other hand, closely related isolates were found. In fact, six pairs of isolates with a cgSNP distance below 10 were found suggesting both regional persistence and spread of clones. In a recent study, high genetic diversity was found in *C. jejuni* collected during processing of caeca and neck skin samples of broilers. These isolates were assigned to ten sequence types, which belonged to seven clonal complexes, based on MLST. ST 257 was prevalent with 58 isolates assigned to it, followed by ST 51 with 25 isolates, ST 10089 with 16 isolates, ST 48 with 13 isolates and ST 50 with 12 isolates (56).

*Campylobacter jejuni* classified by the WHO as a “high priority pathogen” which gives it great concerns due to the emergence of antimicrobial resistance to multiple drugs including fluoroquinolones, macrolides and other clinically relevant classes which limits the alternative treatment for human campylobacteriosis (57).

WGS was used in this study to characterize and predict AMR in this collection of *C. jejuni* isolated of turkey farms from different federal states in Germany. The predicted antimicrobial resistance based on WGS data was concordant with the phenotypic resistance profiles in most cases.

Tetracycline resistance in *Campylobacter* is associated specifically with genes encoding ribosome protection proteins (RPPs) (58). In *Campylobacter*, tetracycline resistance genes can be located both in the chromosomal DNA and on plasmids (59). A clear trend toward an increase in the occurrence of tetracycline and (fluoro)quinolone resistance determinants among *C. jejuni*, linked to the spread of the co-occurring *bla*_OXA−61_ and *tet*(O)-*tet*(O/W/O) genes and the *gyrA* SNP, resulting in the amino acid substitution T86I, was found in the time span from 2001 to date in Europe (60).

The OXA-type β-lactamases confer resistance to the penicillins, although some are also able to cause resistance to cephalosporins and carbapenems. In 2007 European Food Safety Authority (EFSA) considered β-lactams as optional for monitoring at the European union (EU) level (61). A large proportion of *C. jejuni* produce β-lactamases. However, the β-lactamase of *C. jejuni* seems to play a role only in resistance to amoxicillin, ampicillin and ticarcillin (62). In the present investigation, a variety of 13 known β-lactam resistance genes (all *bla*_OXA_ variants) were detected in 58 (87.9%) of the *C. jejuni* isolates, the most prevalent being *bla*_OXA−193_, *bla*_OXA−450_ and *bla*_OXA−605_ (*n* = 31; 47% of each) followed by *bla*_OXA−184_ (*n* = 16; 24.2%). These results were in accordance with a previous study in which two major β-lactamase genes, designated *bla*_OXA−61_ and *bla*_OXA−184_, were prevalent at 62.93 and 82.08% in *C. jejuni* from the poultry and other bird groups, respectively (63).

The finding of this study highlighted that *C. jejuni* is a reservoir for β-lactamase genes that might be transferred to other clinical or environmental bacteria. Thus, screening of *C. jejuni* for such genes may contribute to AMR surveillance in general.

Although point mutations at multiple positions in the *gyrA* gene associated with the resistance to fluoroquinolones in *Campylobacter* have been described (64), the *gyr*A mutation, that results in the amino acid substitution T86I, has been reported as the most prevalent mechanism in *Campylobacter* isolated from animals and humans (64–67). In the present investigation, 93.6% of *C. jejuni* isolates which were phenotypically resistant to both, ciprofloxacin and nalidixic acid, carried this point mutation in *gyrA*. This agrees with previous studies in which this mutation was identified in fluoroquinolone-resistant *C. jejuni* isolated from ruminants and poultry in Spain and Germany by SNP-PCR (68–70). These studies showed that this mutation is present for a long time in *C. jejuni* and still poses concern in isolates from farm animals.

The monitoring of the antimicrobial use in broilers in Germany between 2010 and 2016 showed the highest usage for aminoglycosides followed by fluoroquinolones and a substantial decrease for macrolides and tetracyclines. In turkey flocks, fluoroquinolones were used most frequently, followed by tetracyclines and macrolides. However, in contrast to broilers, the use of aminoglycosides was low in turkeys (30). Mechanisms of aminoglycoside resistance in *Campylobacter* were attributed to enzymatic drug modification (71) and mutations at the ribosomal binding sites (72). Aminoglycoside phosphotransferases (APHs) in *Campylobacter* are mainly encoded by the *aph*(3′)-III, which confers resistance to neomycin and amikacin whereas the *aph*(2″)-Ic gene confers resistance to gentamicin. Aminoglycoside O-nucleotidyltransferases (ANTs) in *Campylobacter* include ANT 6 and ANT 9, which confer resistance to streptomycin and spectinomycin, respectively (71, 73). The *ant*6-I genes encoding aminoglycoside O-nucleotidyl-transferases are widely spread among streptomycin-resistant *C. jejuni* (74). In this study, genes coding for aminoglycoside-modifying enzymes (APHs and ANTs) which confer resistance to amikacin, neomycin, gentamicin, streptomycin and spectinomycin were identified.

PCR was used previously for the determination of streptomycin resistance genes and to recognize *ant*[6]-Ia, *ant*[6]-Ib and other *ant*-like genes (74). Out of 10 phenotypically streptomycin-resistant *C. jejuni*, a gene cluster comprising the genes *sat4, aph*(3′)-IIIa and *aadE* was present in six (60%) connected with aminoglycoside resistance using WGS analysis.

In this study all isolates were phenotypically susceptible to erythromycin despite that five isolates carried a mutation in the gene for the ribosomal protein L22 that resulted in the amino acid substitution A103V, associated with macrolide resistance. A recent study conducted to investigate the genetic basis of antimicrobial resistance in *C. coli* and *C. jejuni* isolated from food animals, poultry processing and retail meat showed that the 23S rRNA (A2075G) mutation was identified only in *C*. *coli* isolates, while *C*. *jejuni* were more likely to harbor the aforementioned mutation in the gene for the L22 protein (23).

As *Campylobacter* are commensal bacteria that are exposed to various antimicrobial agents used in veterinary medicine, additional resistance mechanisms evolved in *Campylobacter* (5). The genes coding for aminoglycosides resistance are usually plasmid-borne (5). A mutation in the *rpsL* gene encoding the ribosomal protein S12 associated with streptomycin resistance was reported only in *C. coli* (72). The contribution of efflux to aminoglycoside resistance in *Campylobacter* is not completely proved, but is likely to be less important than the plasmid-borne genes coding for drug-modifying enzymes (5). Lack of knowledge may explain why resistance-associated genes have not been detected by WGS in four phenotypically streptomycin-resistant *C. jejuni* in the current investigation.

Here, the detection of genetic factors for AMR was performed using different tools and databases (AMRFinder, ResFinder, NCBI) in order not to miss any loci, as there is no single method that might be sufficient for the purpose alone (Table 2). Feldgarden et al. (45) found that AMRFinder appears to be a highly accurate AMR gene detection system (45).

In agreement with the results of this study, other previously published WGS-based studies demonstrated an overall very good concordance between genomic prediction and phenotypic determination of AMR (59, 67, 75, 76). Comparable results were reported by Feldgarden et al. for *C. jejuni*, using the NCBI AMRFinder tool, e.g., with a 98.9% correlation rate (45). A high correlation rate of 97.5% was also found in a recent study from England and Wales (76). There are multiple explanations for possible discrepancies between genotype and phenotype. There might be technical issues, such as low assembly quality (77–79). In this study, high quality of Illumina sequencing was achieved while long read sequencing might improve assembly contiguity (77) which may be helpful especially for plasmid detection and analysis (80). Another technical factor might be incomplete databases, which was counteracted by utilizing several tools. Microbiological factors influencing AMR genotype-phenotype correlation include transcriptional regulation, over-expression and under-expression of genes (e.g., efflux pumps), protein activation or modification or as well as novel resistance genes and mutations may be missed by the currently available databases and search tools (45, 77, 79).

Plasmids played an essential role in the ability of pathogenic bacteria to particularly overcome a new environment and are frequently associated with their virulence. Knowledge of plasmid genetics is significant for the understanding of the evolution and the origin of drug resistance genes (81). In the current investigation, 28 plasmid-borne contigs were detected in sequenced isolates using the Illumina MiSeq. The pTet-like plasmid-borne contig, which carries a *tet*(O) gene, was detected in six phenotypically resistant isolates. It has been reported previously that the tetracycline resistance is not always associated with the presence of pTet-like and in some isolates the gene is located on the chromosome (82). High prevalence of tetracycline resistant *Campylobacter* in chicken was explained in a previous study showed that horizontal transfer of *tet*(O) occurs rapidly and spontaneously without antimicrobial selection pressure between *C. jejuni* isolates in their intestine (83).

## 5. Conclusion

The results of this study emphasize the impact of WGS for in-depth genotyping, screening of virulence, clonality and antimicrobial resistance determinants in *C. jejuni*. In the present study, the antimicrobial resistance genes were mostly identified on the bacterial chromosome, while pTet-like plasmid-borne contigs that harbored the *tet*(O) gene were identified in six *C. jejuni* isolates from different regions (Bavaria and Thuringia) and ST types (122 and 50), suggesting intra-species dissemination of these types of plasmids. Combination of AMR databases are helpful for improving AMR detection in the absence of phenotypic data. Despite the high degree of correlation between phenotypic resistance and genotypes, the phenotypic susceptibility testing is still necessary.

This study revealed a relatively high genetic diversity of *C. jejuni* isolated from turkeys in German flocks while also genetically highly similar isolates were detected. This indicates persistence as well as spread of some *C. jejuni* clones. This finding has to be explored in the future in more detail.

## Data availability statement

The datasets presented in this study can be found in online repositories. The name of the repository and accession number can be found below: European Nucleotide Archive (ENA); PRJEB55640.

## Ethics statement

Ethics approval was not required for the study on animals because the bacterial isolates were taken from the authors' reference laboratory for Campylobacteriosis.

## Author contributions

Conceived and designed the experiments: HE-A, HH, HN, JL, and HMH. Performed the experiments: HE-A, HH, and JL. Analyzed the data: HE-A, HH, HMH, SG-S, and JL. Contributed to the reagents/materials/analysis tools: HE-A, HH, HMH, HT, HN, SS, and JL. Wrote and corrected the manuscript: HE-A, HH, JL, SS, and HMH. Participated in the WGS that were used in the study: HE-A, SG-S, and JL. All authors read and approved the final manuscript.
